# Emergency Laparoscopy for Complex and Trauma Cases: Feasibility and Outcomes in Experienced Surgical Teams

**DOI:** 10.7759/cureus.91138

**Published:** 2025-08-27

**Authors:** Thalia Petropoulou, Aphrodite Fotiadou, Kyriacos Evangelou, Dominika Krasicka, Andreas Polydorou, Manousos Konstantoulakis

**Affiliations:** 1 Second Department of General Surgery, Aretaieion University Hospital, National and Kapodistrian University of Athens, Athens, GRC; 2 Department of Gastrointestinal Surgery, Brighton and Sussex University Hospitals NHS Foundation Trust, Brighton, GBR; 3 Department of Colon and Rectal Surgery, The Euroclinic Hospital of Athens, Athens, GRC; 4 Department of General, Visceral, and Transplantation Surgery, Heidelberg University Hospital, Ruprecht Karl University of Heidelberg, Heidelberg, DEU; 5 Department of Gastrointestinal Surgery, University Hospitals Sussex NHS Foundation Trust, Brighton, GBR

**Keywords:** abdominal trauma, complex surgical emergencies, conversion rate, emergency laparoscopy, experienced surgeon outcomes, general surgery, laparoscopic surgery in trauma, minimally invasive surgery (mis), surgical outcomes

## Abstract

Background

Laparoscopy offers significant benefits over open surgery, including faster recovery, reduced postoperative pain, and fewer complications. However, its feasibility in emergency settings, particularly for complex and trauma-related cases, remains underexplored. Key determinants include surgical expertise, theater readiness, and patient selection.

Methods

This observational, cross-sectional study was conducted over a two-year period in two high-volume university hospitals, one of which is a designated major trauma center. A total of 112 patients undergoing emergency abdominal surgery by a single experienced surgeon were included. Data collected encompassed procedure type, conversion to open surgery, length of stay (LOS), complications, intensive care unit (ICU) admission, and 30-day mortality. Comparative analyses between laparoscopic and open approaches were performed using the Fisher-Irwin exact test.

Results

Among the 112 emergency surgeries, 95 (84.5%) were completed laparoscopically and 17 (15.2%) via open approach. The cohort included category I trauma cases (n=6, 6.3%) and complex conditions such as visceral perforations and bowel obstructions. The median patient age was 75 years, and the median postoperative LOS was five days. Only one laparoscopic case (0.9%) required conversion to open surgery. Diagnostic laparoscopy was also utilized for undifferentiated abdominal pain. Major complications occurred in two (1.7%) patients and required reoperation. ICU admission was necessary in 10 (9%) cases, and the 30-day mortality rate was 1.7% (n=2).

Conclusion

Laparoscopy appears to be a feasible and safe option for emergency abdominal surgery, even in complex and trauma-related cases, when performed by experienced surgeons within adequately equipped settings. The low conversion and complication rates observed in this series reflect the critical role of surgical expertise, team coordination, and appropriate patient selection. While minimally invasive surgery is increasingly becoming standard in many emergency procedures, its successful application in high-risk or complex scenarios remains heavily dependent on operator proficiency. Broader adoption of this approach requires targeted training and further validation through larger prospective studies.

## Introduction

Conventional laparotomy has long been the standard approach for emergency general surgical conditions, particularly in complex cases or trauma [1-4]. While effective, open surgery is associated with prolonged recovery, increased postoperative pain, and higher complication rates [5].

In recent years, minimally invasive surgery (MIS), and specifically laparoscopic approaches, has gained increasing traction in emergency and trauma settings due to its well-documented advantages [1,2,5]. Laparoscopic procedures, which require only small incisions compared to the extensive ones of laparotomy, result in less tissue trauma, reduced postoperative pain, and faster recovery. Patients typically benefit from shorter hospital stays, lower rates of surgical site and hospital-acquired infections, and diminished healthcare costs [6].

Moreover, complications such as incisional hernias, adhesions, and wound infections are significantly less frequent, owing to reduced abdominal wall tension and atraumatic techniques. Laparoscopy also provides enhanced visualization and magnification via high-definition optics, facilitating precise dissection even in challenging anatomy [7]. As a result, it is now considered the standard of care for many elective general surgical procedures [2,8-10] and an optimal tool for diagnostic clarification in uncertain abdominal pathologies [11].

However, in emergency settings, particularly in complex conditions such as perforated ulcers, small bowel obstruction, intra-abdominal sepsis, or traumatic hemoperitoneum, laparoscopic intervention poses unique challenges. These cases often involve distorted anatomy, hemodynamic instability, and a need for rapid decision-making, all of which can limit the feasibility of MIS in less experienced hands.

Successful application of laparoscopy in such emergencies is highly dependent on systemic and institutional factors. These include 24/7 operating room availability, timely access to intensive care unit (ICU) support, and respiratory optimization due to pneumoperitoneum-related cardiopulmonary risks [12-14]. Importantly, surgical team expertise plays a pivotal role. Proficiency in MIS, coordinated teamwork, and familiarity with emergency workflows help avoid intraoperative delays and complications [15]. Trained staff, effective communication, and logistical preparedness, including patient positioning, equipment setup, and postoperative coordination, are essential to safely executing laparoscopy in high-acuity settings.

Despite growing evidence supporting its benefits, the widespread use of laparoscopy in complex or trauma-related emergencies remains limited. As surgical expertise continues to evolve and institutional capacities improve, re-evaluating its feasibility in this subset of patients is timely and warranted.

The present study aims to explore the safety, efficiency, and clinical outcomes of emergency laparoscopic surgery for complex and trauma-related cases in a high-volume, experienced surgical unit, defined as a team led by a board-certified minimally invasive colorectal and general surgeon with extensive laparoscopic expertise, supported by trained perioperative staff accustomed to high-acuity emergency workflows.

## Materials and methods

A prospective, observational, cross-sectional study was conducted across two high-volume university hospitals equipped with advanced laparoscopic facilities, over a two-year period. All consecutive patients undergoing emergency general surgical procedures during on-call shifts by a single, board-certified minimally invasive colorectal and general surgeon were included.

Ethical approval was obtained from both institutional review boards, and written informed consent was secured from all participants. The study was conducted in accordance with the Declaration of Helsinki and reported in line with Strengthening the Reporting of Observational Studies in Epidemiology (STROBE) guidelines for observational studies [16].

All patients undergoing emergency surgery for acute abdominal conditions, including visceral perforations, bowel obstructions, intra-abdominal abscesses, and hemorrhage from blunt trauma, were eligible. Exclusion criteria applied to inguinal hernia cases, which were managed via open surgery as per institutional policy. This decision reflected the prioritization of operating room efficiency during semi-elective on-call hours, aiming to reduce surgical backlogs rather than a limitation in laparoscopic capability. Also, simple acute appendicectomies or cholecystectomies that did not require consultant input were excluded.

Data collected included patient demographics (age and gender), procedure type, conversion to open surgery, length of stay (LOS), postoperative complications, ICU admission, and 30-day mortality. Anesthetic risk was assessed using the American Society of Anesthesiologists (ASA) classification, and operative risk was quantified using the National Emergency Laparotomy Audit (NELA) score [17]. Patients with ASA ≥ IV or NELA ≥ 50%, as well as those experiencing complications, were managed postoperatively in the ICU.

All procedures were performed by the same experienced surgeon with a dedicated, trained team, in fully equipped laparoscopic theaters. This design was chosen to ensure complete consistency in operative technique and perioperative decision-making, thereby allowing the study to reflect the outcomes achievable in the hands of a highly experienced minimally invasive surgeon; however, we acknowledge that this may limit the generalizability of our findings to surgeons with similar training and expertise. Case types included, but were not limited to, diagnostic laparoscopy, adhesiolysis, colectomy, repair of visceral perforations, and control of intra-abdominal hemorrhage. Strict adherence to institutional infection control and safety protocols was maintained.

To minimize bias, several strategies were implemented. Performance bias was mitigated by having all procedures performed by a single experienced surgical team, ensuring consistency in operative technique and perioperative decision-making. Selection bias was reduced through the inclusion of all consecutive eligible cases, with exclusions made solely on the basis of institutional policy. Recall and reporting bias were minimized by using systematically recorded hospital data and objective scoring systems, including the American Society of Anesthesiologists (ASA) classification and the National Emergency Laparotomy Audit (NELA) score. Observer bias was addressed via dual independent data extraction. Institutional bias was limited by conducting the study in two similarly resourced hospitals with comparable laparoscopic capabilities, while technological bias was controlled by including only procedures performed in fully equipped laparoscopic theaters.

Continuous variables are reported as medians, while categorical variables are presented as absolute and relative frequencies. Surgeries were grouped by category (e.g., perforations and colectomies), and comparisons between laparoscopic and open approaches were conducted using the Fisher-Irwin exact test. All p-values are two-tailed, with statistical significance set at p<0.05. Analyses were performed using SPSS version 22.0 (IBM Corp., Armonk, NY).

## Results

Most emergency abdominal operations in our cohort were completed laparoscopically, with a very low conversion rate and favorable postoperative outcomes. Overall complication and mortality rates were low, and the median hospital stay was short.

A total of 112 emergency abdominal surgeries were performed over the two-year study period, including 95 (84.8%) laparoscopic and 17 (15.2%) open procedures (p<0.001). The median patient age was 75 years, with 64 (57.1%) male patients, and a median postoperative length of stay (LOS) of five days. The types of operations performed are detailed in Table 1.

**Table 1:** Emergency surgical procedures by approach (laparoscopic versus open) and associated statistical significance Data are presented as numbers of cases and percentages (number (%)) per group. Statistical significance was assessed using the Fisher-Irwin exact test; as the Fisher-Irwin exact test does not include a test statistic, the Chi-squared with Yates correction test was employed for calculating the corresponding test statistic. *p<0.05 indicates a statistically significant difference between the laparoscopic and open approach for that operation type. ¹One laparoscopic gastric perforation case required conversion to open surgery.

Emergency operation	Laparoscopic (n=95)	Open (n=17)	Test statistic	p-value
Adhesiolysis	4 (4.2%)	-	0.023	1
Ileus	4 (4.2%)	-	0.023	1
Epigastric/internal hernia	6 (6.3%)	4 (23.5%)	3.351	0.0436*
Intussusception	2 (2.1%)	-	0.364	1
Gastric perforation¹	4 (4.2%)	1 (5.9%)	0.094	0.568
Small bowel perforation	4 (4.2%)	-	0.023	1
Hartmann’s procedure for sigmoid colon perforation	10 (10.5%)	6 (35.3%)	5.343	0.0159*
Colectomy with ileostomy	4 (4.2%)	-	0.023	1
Subtotal colectomy	4 (4.2%)	-	0.023	1
Transverse colostomy	2 (2.1%)	-	0.364	1
Defunctioning stoma	6 (6.3%)	-	0.231	0.588
Intra-abdominal washout and drainage	4 (4.2%)	-	0.023	1
Pseudocyst drainage	2 (2.1%)	-	0.364	1
Category I bleeding trauma	6 (6.3%)	4 (23.5%)	3.351	0.0436*
Splenectomy	2 (2.1%)	2 (11.8%)	1.605	0.109
Cholecystectomy	10 (10.5%)	-	0.884	0.355
Diagnostic laparoscopy/appendectomy	23 (40.0%)	-	3.802	0.0006*

**Figure 1:**
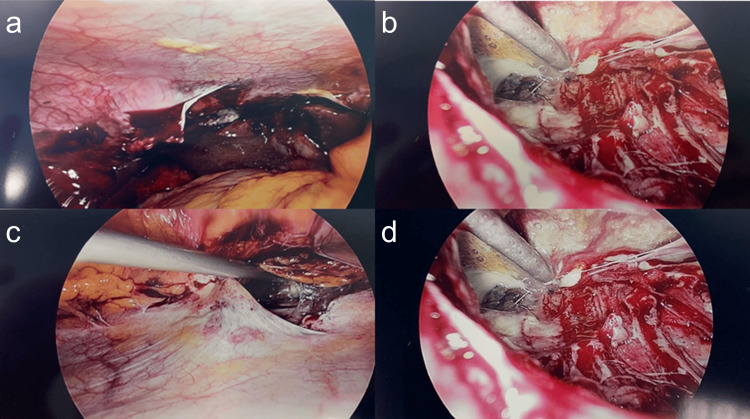
Intraoperative findings of a male patient with acute primary peritonitis managed via diagnostic laparoscopy A large lateral abdominal wall abscess cavity was identified, containing a perforated appendix and over 2 L of purulent fluid and necessitating thorough drainage and lavage. a: Lateral abdominal wall abscess cavity. b: Abdominal wall cavity with pus (drained). c: Lateral abdominal wall cavity with perforated appendix. d: Abdominal wall cavity with pus (drained with normal saline).

All cases of visceral perforation, small or large bowel obstruction, and diagnostic laparoscopy for appendectomy were completed laparoscopically without conversion (Figure 1).

Among trauma cases (n=7, category I), four patients with major hemorrhage and hemodynamic instability underwent immediate open surgery without preoperative imaging. Two of these required open splenectomy due to grade V splenic lacerations. The remaining three trauma patients were managed laparoscopically, with the source of intra-abdominal bleeding successfully identified and controlled using minimally invasive techniques.

Six patients with sigmoid diverticulitis and generalized peritonitis were managed via open surgery. In these cases, both the surgical and anesthetic teams judged that the patients’ frailty and respiratory compromise contraindicated pneumoperitoneum, even at low insufflation pressures.

Only one (0.9%) case required conversion to open surgery. This involved a gastric perforation initially identified laparoscopically, where a safe repair of the posterior wall could not be achieved without conversion. Table 2 summarizes the approaches employed for the above indications.

**Table 2:** Operative management, approach, and notes by indication

Clinical group	Condition	Approach	Patients (number)	Notes
Trauma (category I)	Major hemorrhage: major vessel bleeding	Open surgery: repair	2	No preoperative imaging; required open surgery for major vessel injury
	Major splenic injury (category V)	Open splenectomy	2	No preoperative imaging; required open splenectomy for grade V laceration
	Hemodynamically stable trauma	Laparoscopy	3	Bleeding source identified and controlled laparoscopically
Diverticulitis + peritonitis	Sigmoid diverticulitis + generalized peritonitis	Open surgery	6	Pneumoperitoneum contraindicated due to frailty and respiratory compromise
Gastric perforation	Gastric perforation	Laparoscopy converted to open surgery	1	Conversion due to inability to repair the posterior wall laparoscopically

Two major postoperative complications (1.7%) were observed, both necessitating reoperation. One involved a male patient with necrotizing pancreatitis who deteriorated on postoperative day 2. The second was an elderly, malnourished female with a perforated duodenal ulcer, initially managed with a Graham patch, who developed peritonitis on postoperative day 5.

Ten (8.9%) patients required postoperative ICU care, primarily due to comorbidities or intraoperative findings. The 30-day mortality rate was 1.7% (n=2): one patient died from respiratory failure, and the other from multiorgan dysfunction. Postoperative morbidity, ICU need, and mortality are summarized in Table 3.

**Table 3:** Postoperative morbidity, ICU admissions, and mortality ICU: intensive care unit, POD: postoperative day

Variable	Number of patients (%)	Notes
Major postoperative complications	2 (1.7)	1 necrotizing pancreatitis (male, POD 2, reoperated) and 1 Graham patch failure with peritonitis (female, POD 5)
Postoperative ICU admission	10 (8.9)	Due to comorbidities or intraoperative findings
Mortality	2 (1.7)	1 death from respiratory failure and 1 from multiorgan dysfunction

## Discussion

Our study highlights the feasibility and effectiveness of laparoscopic surgery in emergency settings, particularly in complex and high-acuity cases, when performed by surgeons experienced in minimally invasive surgery (MIS). Notably, 85% of emergency procedures in our cohort were completed laparoscopically, with a low conversion rate of 0.9% and minimal morbidity. These findings underscore the growing role of MIS in acute care surgery and its potential to improve patient outcomes while optimizing healthcare resource utilization. They align with emerging literature suggesting that, in experienced hands and appropriately equipped centers, laparoscopy provides significant advantages even in emergency settings.

Laparoscopy is already widely accepted as the preferred approach for non-complex emergency conditions, such as non-specific abdominal pain (NSAP), and simple gynecologic disorders [1,2,8,18,19]. In these cases, the benefits are well established: reduced hospital stays, faster recovery, and fewer postoperative complications such as wound infections and adhesions [5,20].

However, the scope of laparoscopy extends beyond these standard indications. In expert hands, complex and technically demanding cases, including small bowel obstruction, perforated diverticulitis (Hinchey IV), volvulus, intra-abdominal hernias, and necrotizing infections, can be safely managed laparoscopically. Increasing evidence also supports the use of laparoscopy in hemodynamically stable trauma patients, where it can reduce the rate of non-therapeutic laparotomies and aid in precise intra-abdominal injury assessment [21]. Even the appendectomies in our cohort were for perforated or abscess-forming appendicitis, and most cholecystectomies were for severe or complicated acute cholecystitis, cases that required advanced laparoscopic skills and consultant-level input.

One of the most notable findings in our cohort was the high diagnostic accuracy of laparoscopy, which enabled targeted interventions and helped avoid unnecessary open surgeries. This supports previous reports emphasizing laparoscopy’s dual role in emergency care: as both a diagnostic tool and a definitive therapeutic modality, provided sufficient expertise is available.

Our outcomes also reinforce the idea that surgeon expertise is the most critical determinant of success in emergency laparoscopy [22,23]. Therapeutic procedures, including control of bleeding, resection of necrotic tissue, and repair of visceral injuries, were safely executed laparoscopically, demonstrating that these benefits extend beyond routine cases.

Intraoperatively, the vast majority of procedures were completed laparoscopically without conversion, even in complex scenarios such as perforated diverticulitis, advanced acute cholecystitis, perforated appendicitis with abscess, and hemodynamically stable trauma. The single conversion (0.9%) was due to an inaccessible posterior gastric perforation. Postoperative morbidity was low (1.7% major complications), with only 8.9% of patients requiring ICU admission, primarily for comorbidities rather than surgical complications, and a 30-day mortality of 1.7%. These findings reinforce that, in appropriately selected patients and with experienced MIS teams, laparoscopy can be both safe and effective across a broad spectrum of high-acuity emergency presentations.

Despite its advantages, laparoscopy is not without limitations. Pneumoperitoneum, while essential for visualization, can induce significant physiological changes (elevating intra-abdominal and intracranial pressure, reducing venous return, and causing respiratory compromise (e.g., atelectasis or pneumonia)), especially in elderly or unstable patients [12,24]. Careful patient selection and intraoperative monitoring remain essential to minimize these risks, particularly in those with significant cardiopulmonary comorbidities [2,25].

Beyond patient physiology, the primary barrier to widespread emergency laparoscopy remains institutional and logistical [2,26]. Emergency procedures require rapid decision-making and execution, which may be hampered by limited access to MIS-trained teams, fully equipped laparoscopic theaters, or even basic instrumentation, particularly in resource-limited settings. In such contexts, even technically feasible procedures may default to open approaches due to constraints in personnel or infrastructure.

Moreover, certain clinical scenarios, such as extreme hemodynamic instability or end-stage peritonitis, continue to necessitate laparotomy, despite the expanding role of MIS. Therefore, laparoscopy should be seen as a complementary rather than a universal solution, applied judiciously based on patient factors, surgical expertise, and institutional capacity.

This study supports the concept that, with adequate training, equipment, and team coordination, the boundaries of MIS in emergency care can continue to expand. Advances in resident training programs, team-based operating protocols, and laparoscopic instrumentation are progressively lowering these barriers, allowing broader application in even the most challenging acute cases.

Study limitations

Several limitations must be acknowledged. The small sample size and two-center design may limit generalizability beyond similarly resourced, experienced surgical environments. The non-randomized, observational nature of the study means that causal relationships cannot be definitively established. The relatively small number of open procedures limits the statistical power for direct comparisons between surgical approaches. While the heterogeneity of included procedures reflects real-world emergency practice, it complicates direct comparisons between surgical categories. The absence of long-term follow-up and patient-reported outcomes, including quality of life and satisfaction, is another limitation. Furthermore, institutional policies, such as the exclusion of laparoscopic repair for inguinal hernias, may have introduced bias in case selection.

## Conclusions

This study reinforces that laparoscopic surgery is both feasible and beneficial in emergency general surgical settings, even in complex and trauma-related cases, when performed by experienced MIS surgeons. Its successful application, however, remains highly dependent on surgeon expertise, multidisciplinary team coordination, and institutional readiness. Broader adoption will require targeted investment in surgical training and infrastructure. Future multicenter randomized trials are essential to confirm these findings and define optimal implementation strategies across diverse healthcare environments.
